# Increased percentages of calprotectin and TNF-Α double-positive monocytes in the acute phase of Kawasaki disease

**DOI:** 10.1186/1546-0096-9-S1-O13

**Published:** 2011-09-14

**Authors:** Rolando Cimaz, Giuliana Guggino, Salvatore Accomando, Gabriele Simonini, Ilaria Pagnini, Eiji Ohtsu, Tomisaku Kawasaki, Francesco Dieli, Mahavir Singh, Guido Sireci

**Affiliations:** 1 AOU Meyer Firenze Italy; 2 Università di Palermo, Italy; 3 Kawasaki Disease Research Center, Tokyo, Japan; 4 LIONEX Diagnostics & Therapeutics, Braunschweig, Germany

## Background

The acute phase of KD is characterized by a deficiency of suppressor T cells, marked activation of the immune system and increased secretion of cytokines by immune effector cells. Moreover, it has been shown that myeloid-related protein (MRP-8 and MRP-14) and S100- proteins, the major calcium-binding proteins secreted by activated neutrophils and monocytes, contribute to cause inflammation in acute lesions of KD, and indeed one of the more common hematological alteration in KD is the increase of peripheral blood monocytes. Calprotectin, one of the major calcium-binding proteins, can lead to direct and indirect effects that result not only in inflammation but also in modification of microvascular wall in acute vasculitis syndromes.

## Aim

To study the percentages of “polyfunctional monocytes”, after in vitro exposure to several peptides, as a novel immune correlate of inflammation and endothelial damage mainly occurring in the acute phase of KD**.**

## Methods

We have analyzed the in vitro response of Periperal Blood Mononuclear Cells (PBMCs) to different peptides, i.e. p28, peptide #1, peptide #4 (gpm1 like-molecules derived from S. sanguinis) and peptide #42 (chain A, solution structure of human complement factor H) that could play a critical role in the inflammation of acute phase of KD. We assessed the production of Calprotectin and Tumor Necrosis Factor (TNF)-α in CD14^+^ cells, that might be involved in the endothelial damage. Intracellular Calprotectin was evaluated after stimulation in CD14^+^ cells in patients with acute KD and in age- and sex-matched acute febrile controls. We have enrolled 8 children (5M, 3F, age range 4 months-3 years) affected by Kawasaki disease with cardiac involvement. Only three of them improved with a single cycle of intravenous immunoglobulins, while four needed a second cycle and one also needed intravenous corticosteroids and Infliximab. PBMCs were cultured with selected peptides and cells were fixed, permeabilized, and then stained with anti-human calprotectin-FITC and anti-human TNF-α-PE.

## Results

Patients with acute KD had increased percentages of intracellular Calprotectin in CD14^+^ when stimulated with antigens. On the contrary, age-matched febrile controls did not show any increase of intracellular Calprotectin in CD14^+^ cells after stimulation. The only one patient with acute KD who did not show any increase of intracellular Calprotectin in CD14^+^ cells was the one resistant to traditional therapy and who was subsequently treated with an anti-TNF-α antibody. Additionally, statistically significant higher percentages of double TNF-α^+^ Calprotectin^+^ CD14^+^ were observed (p< 0.05), in PBMCs of patients when compared to febrile controls

## Conclusion

“Polyfunctional monocytes” could represent an important cell subset involved in the generation of acute KD.

